# Physical Activity and Epigenetic Aging in Breast Cancer Treatment

**DOI:** 10.3390/ijms25168596

**Published:** 2024-08-06

**Authors:** Chantalle Moulton, Elisa Grazioli, José Santiago Ibáñez-Cabellos, Arianna Murri, Claudia Cerulli, Monica Silvestri, Daniela Caporossi, Federico V. Pallardó, José Luis García-Giménez, Stefano Magno, Cristina Rossi, Guglielmo Duranti, Salvador Mena-Molla, Attilio Parisi, Ivan Dimauro

**Affiliations:** 1 Unit of Biology and Genetics of Movement, Department of Movement, Human and Health Sciences, University of Rome Foro Italico, 00135 Rome, Italy; c.moulton@studenti.uniroma4.it (C.M.); m.silvestri5@studenti.uniroma4.it (M.S.); daniela.caporossi@uniroma4.it (D.C.); 2 Unit of Physical Exercise and Sport Sciences, Department of Movement, Human and Health Sciences, University of Rome Foro Italico, 00135 Rome, Italy; elisa.grazioli@uniroma4.it (E.G.); a.murri@studenti.uniroma4.it (A.M.); claudia.cerulli@uniroma4.it (C.C.); attilio.parisi@uniroma4.it (A.P.); 3 EpiDisease S.L., Scientific Park, University of Valencia, 46026 Paterna, Spain; j.santiago.ibanez@uv.es; 4 Department of Physiology, Faculty of Medicine and Dentistry, University of Valencia, 46010 Valencia, Spain; federico.v.pallardo@uv.es (F.V.P.); j.luis.garcia@uv.es (J.L.G.-G.); 5 INCLIVA Health Research Institute, INCLIVA, 46010 Valencia, Spain; 6 Consortium Center for Biomedical Network Research on Rare Diseases (CIBERER), Institute of Health Carlos III, 46010 Valencia, Spain; 7 Center for Integrative Oncology, Fondazione Policlinico Universitario A. Gemelli IRCCS, 00136 Rome, Italy; stefano.magno@policlinicogemelli.it (S.M.); cristina.rossi1@guest.policlinicogemelli.it (C.R.); 8 Unit of Biochemistry and Molecular Biology, Department of Movement, Human and Health Sciences, University of Rome Foro Italico, 00135 Rome, Italy; guglielmo.duranti@uniroma4.it; 9 Department of Physiology, Faculty of Pharmacy, University of Valencia, 46100 Burjassot, Spain

**Keywords:** DNA methylation, epigenetic clock, exercise, telomere

## Abstract

Biological age, reflecting the cumulative damage in the body over a lifespan, is a dynamic measure more indicative of individual health than chronological age. Accelerated aging, when biological age surpasses chronological age, is implicated in poorer clinical outcomes, especially for breast cancer (BC) survivors undergoing treatments. This preliminary study investigates the impact of a 16-week online supervised physical activity (PA) intervention on biological age in post-surgery female BC patients. Telomere length was measured using qPCR, and the ELOVL2-based epigenetic clock was assessed via DNA methylation pyrosequencing of the *ELOVL2* promoter region. Telomere length remained unchanged, but the ELOVL2 epigenetic clock indicated a significant decrease in biological age in the PA group, suggesting the potential of PA interventions to reverse accelerated aging processes in BC survivors. The exercise group showed improved cardiovascular fitness, highlighting PA’s health impact. Finally, the reduction in biological age, as measured by the ELOVL2 epigenetic clock, was significantly associated with improvements in cardiovascular fitness and handgrip strength, supporting improved recovery. Epigenetic clocks can potentially assess health status and recovery progress in BC patients, identifying at-risk individuals in clinical practice. This study provides potential and valuable insights into how PA benefits BC survivors’ health, supporting the immediate benefits of a 16-week exercise intervention in mitigating accelerated aging. The findings could suggest a holistic approach to improving the health and recovery of post-surgery BC patients.

## 1. Introduction

Biological age is the gradual accumulation of damage in the body over the lifespan, leading to a decline in system reserve and increased risk of age-related diseases, disability, and mortality, [1,2,3]. Biological age is a more dynamic and individualized measure as compared to chronological age and can be used to assess an individual’s susceptibility to certain diseases and their overall health. Various aspects, including genetic, environmental, and lifestyle factors, may contribute to biological age. Accelerated aging, where biological age overtakes chronological age, has been implicated in heightened risks of cardiovascular disease, diabetes, dementia, and cancer [1,2,3].

Among the plethora of contributors to accelerated biological aging, cancer and its treatments stand out, particularly in the context of breast cancer (BC) recovery. BC, a group of diseases with profound implications, commands attention not only due to its prevalence but also for the challenging journey of recovery that it requires and highlights the importance of exploring innovative approaches to enhancing recovery and survivorship. In BC patients, measurement of biological age has revealed accelerated biological aging as compared to non-cancerous age-matched counterparts [4], where the adverse effects of chemotherapy and radiation therapy on hallmarks of aging, such as telomere attrition, stem cell exhaustion, epigenetic drift, and DNA damage, accentuate the accelerated aging process in cancer survivors [2,4,5]. This accelerated aging not only jeopardizes functional outcomes but also poses increased health risks for cancer survivors [6,7,8].

While the many molecular pathways and biological processes contributing to increased biological age in cancer recovery are still not fully understood [4,5,9], certain markers associated with accelerated aging, such as telomere length (TL) and epigenetic clocks, offer a promising avenue for assessing recovery progress and prognosis in BC patients [10,11,12].

Telomeres are genomic complexes located at the end of chromosomes that protect DNA during DNA replication [13,14]. While TL can be influenced by certain lifestyle factors, it is associated with age-related diseases, lifespan, and cancer, specifically with increased cancer malignancy, poor prognosis, and risk of metastasis and death [15,16]. When TL is critically short, resulting DNA damage may lead to one of two outcomes: (1) the pathological cell undergoes apoptosis, increasing senescence which is linked to aging; or (2) the non-functional cell continues to produce damaged proteins, increasing the risk of cancer development [17,18]. When looking to relationships between TL and levels of physical activity (PA), the current literature indicates a possible relationship between higher PA levels and longer skeletal muscle or leukocyte TL, with an increased cardiovascular fitness or cardiovascular training associated with increased TL, however, results remain inconclusive and may be attributed to differences in exercise protocols used [19,20].

Epigenetic clocks are also gaining attention in the measurement of biological age, with interests in health span, disease recovery, and prognosis, as well as in female reproduction timelines and pregnancy outcomes [21,22,23,24,25]. High biological age is associated with a higher risk of disease and death, while a younger biological age is linked to a lower risk and a better health status. Most epigenetic clocks are based on DNA methylation (DNAm) patterns, which is the addition of a methyl group to the 5′ position of the cytosine pyrimidine ring on cytosine-phosphate-guanine (CpG) dinucleotides, constituting a flexible genomic parameter that can alter gene expression without altering the DNA sequence. These patterns change with age and health and can be used to predict a biological age [21,26].

Several epigenetic clocks have been employed in cancer research to unveil the intricate relationship between DNAm patterns and biological age. For instance, the Horvath Clock, a multi-tissue clock utilizing 353 CpG sites, has been extensively utilized to assess the biological age in various cancer contexts [21,27,28]. Hannum’s Clock, based on 71 CpG sites, was initially developed using blood samples and has proven valuable in unravelling age-related alterations in cancer patients [21,29,30]. DNAm PhenoAge, calculated based on 513 CpG sites, has also demonstrated its utility in understanding the biological age of individuals in the context of cancer and age-related conditions [31]. Moreover, the Zbieć-Piekarska age predictor, which measures 7 CpGs in the *ELOVL2* (Elongation Of Very Long Chain Fatty Acids-Like 2) gene promoter, where the DNAm patterns are very strongly associated with age, has been applied to evaluate age-related changes in different physiopathological conditions [32,33,34].

Although not yet incorporated into clinical practice, these diverse epigenetic clocks could contribute to a comprehensive understanding of the biological age landscape in cancer, potentially paving the way for targeted therapeutic interventions [10,12,34].

PA significantly benefits cancer patients by mitigating treatment side effects. PA combats muscle wasting, enhances cardiorespiratory fitness, and reduces negative symptoms, including pain, anxiety, nausea, and sleep disturbances, thereby improving health-related quality of life (QoL) and physical functioning [35,36]. Recent studies also show that PA lowers stress and depression in advanced-stage cancer patients while improving pain, fatigue, respiratory issues, and sleep, making PA safe and essential for comprehensive cancer care, boosting patient autonomy and well-being [37,38,39,40,41,42].

Furthermore, PA is one of the limited interventions that has the ability to improve lifespan and health span by preventing age-related diseases and early death [43]. PA causes a plethora of biochemical stimuli (including oxidative stress, immune responses, and changes in the metabolic flux of key metabolic pathways) in various tissues and, in turn, impacts gene expression, mediating epigenetic modifications such as DNAm [44,45].

Studies have shown that both acute and chronic exercise have the capacity to significantly impact DNAm, in a highly tissue- and gene-specific manner [46,47]. Furthermore, on a deeper molecular level, PA has been found to specifically modulate biological age, as measured by various DNAm clocks [24,48], with the potential to mitigate the aging process. Scientific investigations into the effects of PA on DNAm and epigenetic clocks has produced the DNAmFitAge, a DNAm clock linking exercise to the epigenome, highlighting the association between regular physical activity and slower aging [49]. Furthermore, cardiovascular fitness in older men was inversely correlated with biological age based on the DNAm-Age Acceleration clock [50].

To date, the relationship between PA, DNAm, and the epigenetic clock is intricate and not fully understood, possibly dependent on factors like exercise characteristics (i.e., type, frequency, intensity, and duration), tissue specificity, and individual genetic variations [44,46,48,49]. Although some evidence suggests the potential of regular PA to positively influence the DNAm clock and impede biological aging, further research is crucial for a comprehensive understanding of these effects [24].

Given the paucity of studies measuring the interplay among BC, the medical treatment, biological age, and the possible impact of PA, the primary objectives of this preliminary study are to gather initial insights on the effect of an online supervised exercise training program on biological age in post-surgery female BC patients undergoing medical treatment. In particular, we measured both (1) relative TL and, for the first time, (2) the methylation of specific DNA methylation sites in the *ELOVL2* gene, which set the basis for an epigenetic clock as markers of aging and health, to identify any changes in biological age. Moreover, to quantify the effects of this PA program on physical fitness and function, we analyzed different functional parameters (i.e., balance, flexibility, strength, and cardiovascular fitness), QoL, and body mass index (BMI). Finally, to elucidate any possible relationships between changes in physical functions (i.e., cardiovascular fitness and strength) and changes in biological age, we compared our results by using a correlation matrix and simple linear regressions analysis.

## 2. Results

### 2.1. Participant Baseline Characteristics

As shown in Table 1, at baseline (PRE), no significant differences were observed between BC patients belonging to the CG and EG in participants’ chronological age (CG vs. EG: 53.00 ± 6.65 yrs. vs. 50.21 ± 5.47 yrs., *p* = 0.2852), BMI (CG vs. EG: 22.76 ± 2.30 kg/m^2^ vs. 27.16 ± 6.11 kg/m^2^, *p* = 0.0747) and physical activity levels (CG vs. EG: 834.6 ± 596.0 vs. 854.3 ± 587.5 MET-min/week, respectively *p* = 0.695). Both experimental groups were also homogenous for the type of surgery and medical treatment.

### 2.2. Physical Activity Improves Functional Parameters in Breast Cancer Patients

To evaluate changes in physical function and performance, multiple analyses were performed to measure parameters for physical fitness, strength, mobility, and feelings of discomfort. All results are listed in Table 2. Of these parameters, hand grip strength (HG) was significantly decreased in the left side in CG at the end of the experimental period (PRE vs. POST: 24.12 ± 2.204 vs. 20.80 ± 0.4967, *p* = 0.0019); the sit-to-stand test (STS) showed a significant increase in EG after the exercise intervention (PRE vs. POST: 18.00 ± 3.843 vs. 21.29 ± 4.906, *p* = 0.0413); the sit and reach test (STR) significantly increased in EG (PRE vs. POST: −0.5714 ± 9.277 vs. 4.929 ± 8.265, *p* = 0.0035); the scratch test (ScT) significantly decreased in EG on both the right side (PRE vs. POST: 27.23 ± 9.418 vs. 20.92 ± 7.609, *p* = 0.002), and the left side (PRE vs. POST: 27.46 ± 5.897 vs. 22.77 ± 5.988, *p* = 0.0002); as a measure of physical fitness, the 6-min walk test (6MWT) significantly increased in EG (PRE vs. POST: 597.1 ± 67.05 vs. 628.6 ± 63.20, *p* = 0.0025); and as a measure of physical discomfort, the Borg scale was measured to be significantly lower in EG at the end of the experimental protocol (PRE vs. POST: 2.67 ± 0.4875 vs. 1.571 ± 1.072, *p* = 0.0127). All other measures were not significantly changed at the end by the experimental period.

### 2.3. Physical Activity Does Not Affect Relative Telomere Length in Breast Cancer Patients

**Figure 1:**
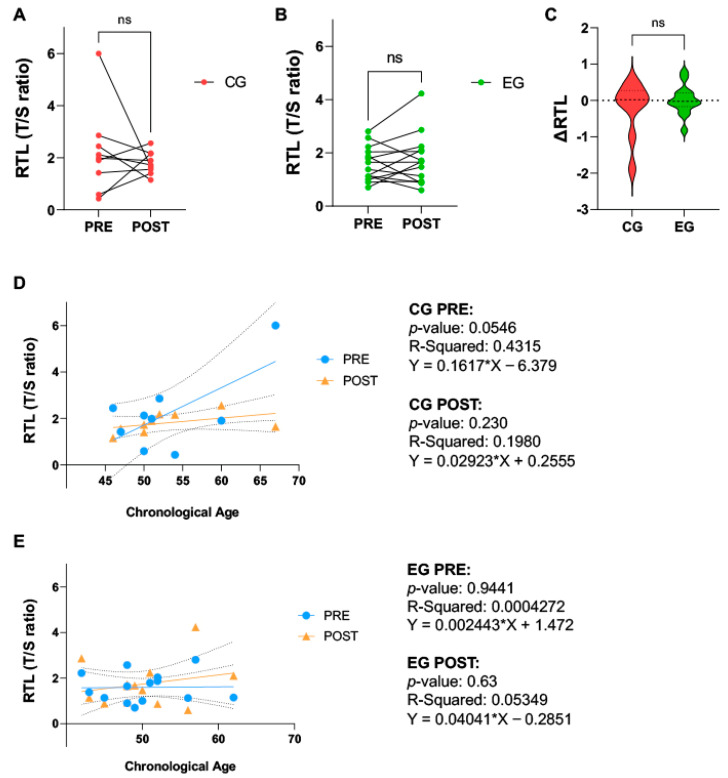
Results of relative telomere length (RTL) analyzed from DNA extracted from whole blood, obtained at the beginning (PRE) and at the end (POST) of the experimental protocol in female breast cancer patients, randomly assigned to the Control Group (CG, n = 9), where all subjects received the usual cancer treatments, or the Exercise Group (EG, n = 14), where volunteers were additionally included in a 16-week online training program. RTL at PRE and POST of (**A**) CG and (**B**) EG. (**C**) Difference in RTL. Regression showing the association of chronological age and RTL in (**D**) CG and (**E**) EG. EG, Exercise Group; CG, Control Group. ns: not significant.

Relative telomere length (RTL) remained unchanged at the end of the experimental period in either group (EG: PRE vs. POST, 1.595 ± 0.6459 vs. 1.744 ± 0.9550, *p* = 0.4631; CG: PRE vs. POST, 2.191 ± 1.637 vs. 1.805 ± 0.4369, *p* = 0.5224) (Figure 1A,B). No significant differences were seen in the changes in RTL over the experimental period, when comparing the two groups (EG vs. CG, 0.0066 ± 0.4029 vs. −0.2141 ± 0.7564, *p* = 0.4379) (Figure 1C). No significant associations of RTL with chronological age were identified through simple linear regressions in either CG or EG, PRE or POST (Figure 1D,E).

### 2.4. Health-Related Quality of Life

Data regarding health-related QoL were measured with the EORTC QLQ-C30 (Table 3). The EG showed an increase in physical function after training (PRE vs. POST, *p* = 0.040). Moreover, the other subscales (emotional function, cognitive function, social function, and global health) registered small increases, though not significant. In the CG, no significant differences were observed for any of the parameters from the EORTC QLQ-C30 (*p* > 0.05) at the end of the experimental period (Table 3).

### 2.5. Physical Activity Improves ELOVL2-Based Epigenetic Clock in Breast Cancer Patients

**Figure 2:**
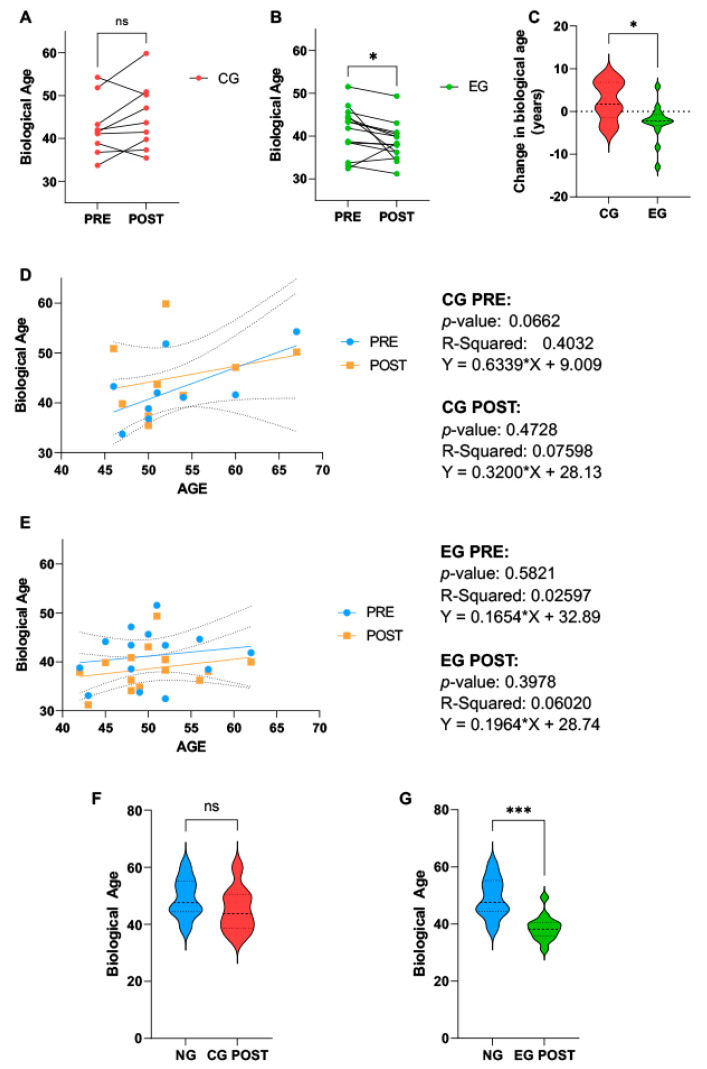
Results of biological age analyzed from DNA extracted from whole blood, obtained at the beginning (PRE) and at the end (POST) of the experimental protocol in female breast cancer patients, randomly assigned to the Control Group (CG, n = 9), where all subjects received the usual cancer treatments, or the Exercise Group (EG, n = 14), where volunteers were additionally included in a 16-week online training program. Biological age at PRE and POST of (**A**) CG and (**B**) EG. (**C**) Change in biological age in number of years. Regression showing the association of chronological and biological age in (**D**) CG and (**E**) EG. Finally, the comparison between a healthy, age-matched non-cancerous group (NG, n = 16) with (**F**) CG POST and (**G**) EG POST, to compare the effects of medical treatments and physical activity on biological age. * *p* < 0.05; *** *p* < 0.001. EG, Exercise Group; CG, Control Group. ns: not significant.

As measured by the ELOVL2 epigenetic clock, there was a significant decrease in biological age in EG (PRE vs. POST, 41.20 ± 5.611 vs. 38.60 ± 4.37, *p* = 0.0409), whereas in CG, no significant changes were found (PRE vs. POST, 42.61 ± 6.64 vs. 45.09 ± 7.72, *p* = 0.140) (Figure 2A,B). When comparing the change in biological age at the end of the experimental period, the difference in biological age was significantly lower compared to CG (EG vs. CG, −2.6 ± 4.286 vs. 2.484 ± 4.546, *p* = 0.0163) (Figure 2C). Simple linear regression identified no significant associations between chronological age and biological age in both groups (Figure 2D,E), although, there is a trend which places POST EG participants at a lower biological age than at PRE (EG: PRE vs. POST, Y = 0.1654*X + 32.89 vs. Y = 0.1964*X + 28.74) (Figure 2E).

In the non-cancerous group (NG), the biological age was 49.07 ± 6.834. To evaluate the effects of medical treatment and PA on the biological age as compared to NG, *t*-tests between NG and the POST of CG and EG were performed, respectively. NG and CG POST were not significantly different (NG vs. CG POST: 49.07 ± 6.834 vs. 45.09 ± 7.72, *p* = 0.2167), whereas, when comparing NG with EG POST, the biological age of EG POST was significantly lower (NG vs. EG POST: 49.07 ± 6.834 vs. 38.60 ± 4.37, *p* < 0.0001) (Figure 2F,G).

### 2.6. Correlation and Regression Analysis

To evaluate possible associations among parameters of physical function and markers of biological aging, as well as to describe how they are numerically dependent on markers of biological age, a correlation matrix as well as linear regression analysis were conducted using the change (either the difference or fold change) of each variable. The analyses were performed using the overall change in each variable, excluding age which was considered unchanged during the protocol, without distinguishing between the two groups.

**Figure 3:**
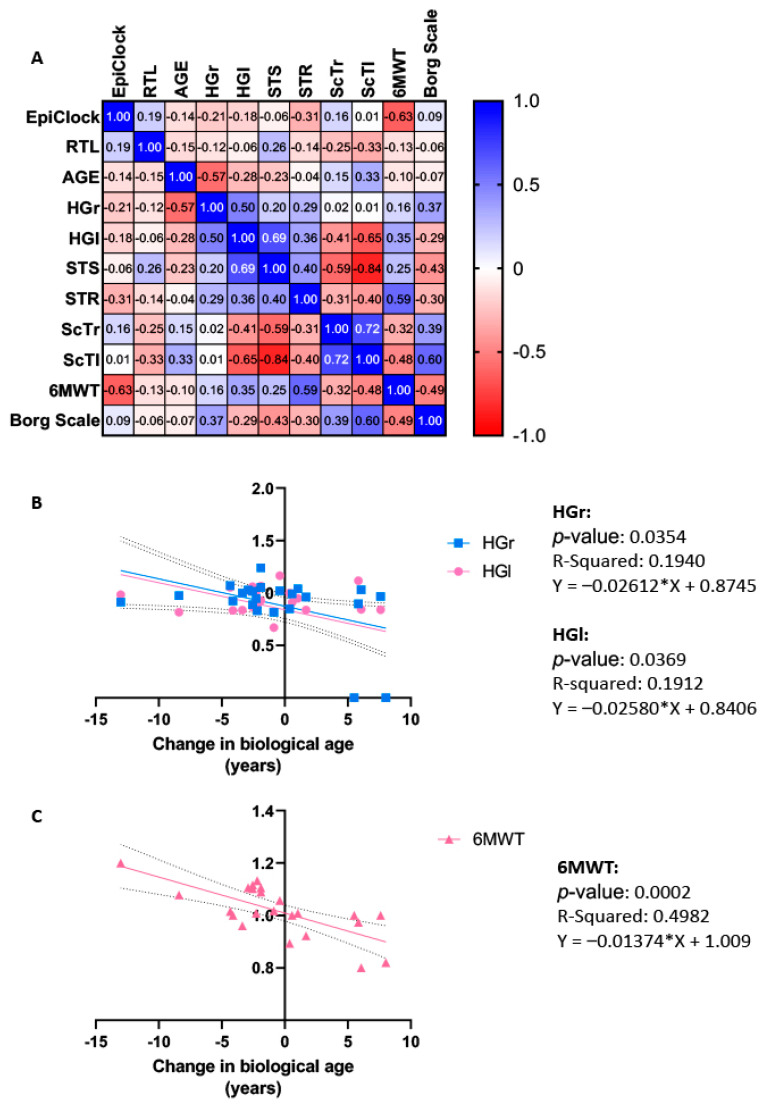
(**A**) Heat map representation of a correlation matrix showing correlations between changes in biological age, relative telomere length, and functional outcomes and physical performance measures; and linear regression analysis between changes in biological age and functional outcomes including (**B**) changes in cardiovascular fitness (6MWT) and (**C**) hand grip strength of both the right (HGr) and left (HGl) sides, measured in female breast cancer patients assigned to either the Control Group (CG, n = 9), where all subjects received the usual medical treatments, or Exercise Group (EG, n = 14), where they were additionally included in a 16-week online supervised exercise training intervention. (----------) 95% confidence band. R square represents the contribution of the independent variable to a clinical parameter (dependent variable) in the univariate analysis. A low *p*-value (<0.05) indicates a significant relationship between variables. EpiClock, ELOVL2-based epigenetic clock; 6MWT: 6-min walking test; HGr, Hand grip strength right; HGl, Hand grip strength left; STS, sit-to-stand test; STR, sit and reach test; ScTr, Scratch test right; ScTl, Scratch test left.

As represented by Figure 3A, Spearman’s correlation analysis revealed that changes in biological age showed a significant strong negative correlation with changes in 6MWT (r = −0.631, *p* = 0.001), while no other measures of physical function were correlated with ELOVL2-based **e**pigenetic **c**lock or RTL (*p* > 0.05). However, in terms of parameters of physical function, changes in the 6MWT were moderately correlated with changes in STR (r = 0.589, *p* = 0.005), and moderately correlated with both ScTl (r = −0.477, *p* = 0.039) and Borg scale (r = −0.493, *p* = 0.023). HGr was strongly correlated with age (r = −0.571, *p* = 0.004) and moderately correlated with changes in HGl (r = 0.496, *p* = 0.016). Changes in HGl were also strongly correlated with both changes in ScTl (r = −0.645, *p* = 0.003) and STS (r = 0.694, *p* = 0.0002). Changes in STS were further moderately correlated with ScTr (r = −0.586, *p* = 0.008) and very strongly correlated with ScTl (r = −0.842, *p* = 0.00001). Finally, changes in ScTl were strongly correlated with changes in ScTr (r = 0.715, *p* = 0.001) and moderately correlated with the Borg scale (r = 0.595, *p* = 0.007). No other correlations were significant (*p* > 0.05).

The use of univariate analysis revealed biological age as a significant independent variable in the context of HGr, HGl, and 6MWT, accounting for 19.4%, 19.1%, and 49.8% (Figure 3B,C), respectively. All other analyses using univariate analysis were not significant (*p* > 0.05).

## 3. Discussion

Our results highlight the beneficial effects of PA in post-surgery BC patients undergoing medical treatment, demonstrating a potential reduction in biological age. This reduction is evident in the reversal of the ELOVL2-based epigenetic clock, which, in turn, is associated with improved physical outcomes. This association is supported by significant correlations with increased hand grip strength and improved cardiovascular fitness.

The reversal of the ELOVL2 epigenetic clock, a measure of age-related changes at the molecular level [31], observed in the EG, indicates a significant decrease in biological age within our cohort of BC patients following a 16-week exercise intervention. This finding underscores the capacity of exercise interventions to mitigate and reverse accelerated aging processes, particularly in this vulnerable population.

Cancer is a distinctive disease as its own treatment often causes damage and accelerated aging [4,5,7], and the ability to assess biological aging becomes paramount for understanding BC patients’ ability to manage cancer therapy, their clinical and functional outcomes, and overall cancer progression [6,7,8].

TL, a prevalent marker of aging, revealed no significant changes in response to medical treatment or PA at the end of the experimental period; this finding aligns with the evolving understanding that adaptations in TL may require more extended interventions for noticeable changes [51,52]. The lack of significant changes in TL in response to medical treatments or PA in our BC patient sample may stem from interindividual differences or the relatively short duration of our intervention. Extensive literature suggests that medical treatments, such as chemotherapy, can shorten telomeres, associated with accelerated aging, while PA may lengthen or maintain TL [14,51,53]. While we saw no significant changes at the end of the 16-week period, a longer intervention may uncover positive effects on TL due to PA, consistent with findings from longer-duration studies [19]. Furthermore, the age range in our study populations may be too narrow to reveal any trends in TL with chronological age due to potential biases that would be avoided against in larger populations with a wider age range. However, it is also notable that these potential trends might be eclipsed or altered due to BC, as is possible in pathological conditions, where factors such as the cell proliferation, inflammation, and disease-specific mechanisms can complicate the relationship between age and TL [54]. In addition, hormonal changes associated with menopause status may also have an effect on TL analyzed in blood samples [55]. To this end, a comprehensive understanding of the interplay between menopause status, age ranges, and pathological conditions is crucial for accurately interpreting TL. However, on the contrary, limited research suggests that epigenetic clocks, especially the ELOVL2-based epigenetic clock, may operate independently of menopause status [56], emphasizing their potential robustness and suitableness for BC clinical practice.

The reduction in biological age, as measured by the ELOVL2-based epigenetic clock, as seen in the EG, supports the literature which confirms a beneficial effect of PA on BC recovery. Though the changes in CG were not significant, we can hypothesize that, given a longer study period, this trend of the CG biological age to increase, could potentially be significant due to the stress of medical treatment, known to accelerate biological aging [5]. A reduction in the biological age in EG would propose a reduced mortality with increased survival, as well as an improved overall health and lifespan [25]. In fact, the significant decrease in biological age in EG at the end of the intervention, to reach levels lower than that of HG, a representation of a similar age-matched population without pathologies, suggests the capacity of PA to improve health, reducing all risks associated with age-related diseases [25]. The immediate changes observed in biological age in EG due to 16 weeks of PA intervention, giving an immediate reflection of beneficial effects on aging and cancer compared to the slower adaptation process of TL, suggest that PA may exert faster and more tangible effects on the molecular level, contributing to BC patient recovery. While the beneficial effect of PA on improved cardiovascular fitness and physical functioning in BC patients has already been established in the literature [47,57], improved functional performance was associated with a decrease in biological age, as demonstrated by the significant associations between reduced biological age and enhanced hand grip strength and cardiovascular fitness measured by the 6MWT. Improved hand grip strength and cardiovascular fitness, as associated with decelerated biological age, contribute to enhanced overall health, potentially leading to a faster and more robust recovery process. This could reduce medical expenses associated with extended hospital visits and increased medication reliance. Not only do these correlations highlight the potential of using epigenetic clocks in clinical practice to evaluate patients’ progress, identify at-risk individuals, and ultimately enhance recovery, but also reveal the interplay between countless PA-modulated pathways on the molecular level, translating to a systematic level to improve health and function [25,45,47,57].

Finally, our study has shown a significant improvement only in the physical function subscale of the EORTC-C-30. Similarly to our previous research [57], the exercise protocol was able to improve QoL, although there were only slight differences in their respective domains. This is likely due to differences in the characteristics of the patients, the exercise, and the measurement methods when compared with other published results.

To date, further research is certainly needed to fully understand the effects of PA on DNAm age, and their interplay in cancer recovery, although current evidence, as well as our results, suggests that regular PA can have beneficial effects on the DNAm clock and potentially slow the process of biological aging, with little to no studies finding a harmful increase in systemic biological age due to PA [24].

## 4. Future Perspectives, Limitations, and Clinical Considerations

It is crucial to acknowledge the challenges associated with the accessibility and cost of DNAm analysis in clinical practice. Many epigenetic clocks demand extensive CpG site analyses, requiring substantial resources and expenses. In contrast, the ELOVL2-based DNAm clock requires fewer resources, as it is considering only a minimal number of CpGs making the assessment of biological age more practical and affordable in a clinical setting. The ELOVL2 clock has also shown consistent performance across different populations, indicating its robustness and generalizability. This may provide more information about patients’ recovery and identify at-risk patients for poorer clinical outcomes, enabling tailored and more specialized care. Epigenetic clocks are also constantly evolving and being “fine-tuned” and developed, and the further evolution of this technology would make this a more easily accessible tool to use in clinical practice [26].

The mechanisms behind the changes in biological age due to PA are still poorly understood and more research is needed to understand the beneficial effect of PA. Moreover, understanding the biological mechanisms of accelerated aging and developing strategies to mitigate its consequences remains a crucial avenue for improving the QoL for cancer survivors. Also, longitudinal and follow-up studies should be carried out to analyze how long these changes induced by PA last, or if continued PA throughout the lifespan is necessary to maintain them. Furthermore, future research should explore, in larger cohorts and longer interventions, the optimal intensity, duration, and modality of exercise interventions to maximize the benefits observed in our study.

Although this preliminary study provides valuable insights into the potential benefits of an online supervised exercise training program on biological age in post-surgery female BC patients, several limitations should be considered.

As such, this study has been conducted with the aim to conduct an initial investigation to gather basic information, test hypotheses, or evaluate the practicality and viability of a larger, more comprehensive study. Indeed, we include a small sample size which is somewhat heterogenous in nature with various clinical characteristics, where the patients may receive slightly different medical treatments, have different stages of BC, or may have possible differences in cancer genotype. While this could explain the interindividual differences we observed, on the other hand, focusing on a smaller group allows for the collection of rich, detailed data, providing valuable insights that are essential for exploratory research. This depth of information is invaluable in laying the groundwork for future studies.

Other possible limitations include the lack of a healthy age-matched group that performed the physical activity protocol and information regarding menopause status of the patients included in the study; potentially too short of an experimental period to measure TL; and the lack of a social meeting equivalent in the CG to control for the social aspect of our exercise intervention in the EG. However, despite these limitations, this preliminary study is a prospective and supervised physical activity intervention study where our results may contribute to the growing body of literature on the interplay between lifestyle changes, molecular aging markers, and cancer recovery.

## 5. Materials and Methods

### 5.1. Ethical Approval

This study received ethical approval from the Ethics Committee of the University of Rome “Sapienza” under the reference RIF.CE: 5451_2019. Informed consent was obtained from all participants, and the study was conducted in compliance with the most recent version of the Helsinki Declaration (October 2013). This study forms a part of the ongoing EPIAF-study (Epigenetic and Physical Activity) launched by the University of Rome “Foro Italico”, A. Gemelli University Hospital Foundation IRCCS in Rome, Italy.

### 5.2. Study Design

This preliminary study is part of the ongoing EPIAF project, a randomized controlled trial which lasts 16 weeks. All information about the study design, selection criteria, patient assessments, and randomization procedures can be found as reported previously [47,57]. Briefly, the EPIAF study is a randomized controlled exercise study involving female volunteers diagnosed with BC. The participants were randomly divided into a Control Group (CG, n = 9) and an Exercise Group (EG, n = 14). All patients (CG and EG) received standard anticancer medical treatment as administered by the hospital, but EG performed an additional exercise training intervention. As previously described [57], various assessments were conducted, including medical examinations, food diaries, anthropometric measures, and evaluation of physical activity levels. Functional capacity parameters and patient-reported outcomes were assessed at the beginning and at the end of a 16-week experimental period. In addition, a non-cancerous group (NG), a sample of 40–60-year-old non-cancerous females (n = 16), who had already been analyzed for biological age using the ELOVL2-based epigenetic clock, was included to serve as a as referent biological age of age-matched healthy females measured by the ELOVL2-based epigenetic clock. In this study, a small sample size is justified due to its exploratory nature and helps to minimize potential risks and burdens for participants.

### 5.3. Exercise Training Program

As previously reported [47,57], the exercise training intervention, supervised by a qualified kinesiologist from the University of Rome “Foro Italico”, was conducted online twice per week for 1 h per session over a 16-week period, totaling 32 sessions. Briefly, all sessions were individualized to each participant and their fitness status and fatigue levels (measured by International Physical Activity Questionnaire—IPAQ, maximal heart rate, and the use of the Borg scale), consisting of a warm-up, resistance exercises, aerobic training, and a cool-down phase. The detailed description of the training phases can be found as previously reported [57].

### 5.4. Blood Sampling and DNA Extraction

From each participant, fasted blood samples were collected in EDTA tubes via the antecubital vein at the beginning (PRE) and end (POST) of the 16-week experimental period, and aliquoted and stored at −80 °C until further analysis. Later, genomic DNA was extracted from 100 μL of whole blood using the DNeasy Blood & Tissue Kit (Qiagen, Hilden, Germany) following the manufacturer’s instructions. DNA was quantified using a NanoDrop 2000 (ThermoFisher Scientific, Waltham, MA, USA).

### 5.5. Measurement of Relative Telomere Length

Measurement and quantification of RTL were carried out following the protocol described by Joglekar and colleagues [58] using a quantitative polymerase chain reaction (qPCR) to obtain the ratio of the telomere product versus a single copy gene (T/S ratio) with human β-hemoglobin (Hbg) serving as the single copy gene. An amount of 25 ng of DNA was loaded into each well, with each sample analyzed in triplicate along with water/no template control (NTC) and a reference/control DNA (blood sample from a non-study sample/cell line DNA). The qPCRs were run using the QuantStudio™ 5 Real-Time PCR System (ThermoFisher Scientific). RTL was calculated using the comparative 2^−ΔΔCt^ method: relative T/S ratio = 2^−ΔΔCt^ where ΔΔCt = (Ct Telomere − Ct Hbg) sample DNA − (Ct Telomere − Ct Hbg) control DNA.

### 5.6. Measurement of ELOVL2-Based Epigenetic Clock

An amount of 500 ng of DNA was bisulfite converted using the EZ DNA Methylation Kit (Zymo Research, Freiburg, Germany), according to manufacturer’s instructions. A PCR was then performed using 3 µL of bisulfite-converted DNA to amplify the region of interest in the *ELOVL2* promoter. Next, Agingmetrix test (EpiDisease SL, Valencia, Spain), based on methylation status of 9 CpGs within the *ELOVL2* promoter region, was used to calculate the biological/epigenetic age. Briefly, bisulfite-converted DNA was amplified by PCR using proprietary PCR primers. Each PCR amplification region was analyzed by Pyrosequencing using Pyromark Q24 (Qiagen). The methylation percentages at each CpG were calculated according to a proprietary algorithm for estimation of biological age. All these data obtained at the end of the study period were compared with those obtained with the same epigenetic clock from a healthy, non-cancerous population of age-matched females (NG, non-cancerous group) (female, n = 16, age 40–60).

### 5.7. Statistical Analysis

All statistical analyses were performed using GraphPad Prism software 10.0 (GraphPad Software, San Diego, CA, USA). Quantitative variables were expressed as mean (±SD), and normality tests were conducted to assess data distribution. For normally distributed, continuous variables, a Student’s *t*-test was used. A correlation matrix was used to demonstrate the strength of associations between the changes in biological age, RTL, and various functional outcomes and physical performance measures. Additionally, simple linear regression analyses were also carried out to examine the relationships between the changes in all variables and the contribution of biological age as an independent variable based on the correlation data. In all instances, a *p*-value ≤ 0.05 was considered significant.

## 6. Conclusions

In conclusion, our findings support the integration of a tailored, individualized PA program, alongside the adjuvant medical treatments of post-surgery BC patients, supported by the potential to reduce ELOVL2-based biological age and enhance functional capacities. Analysis of the biological age may allow for personalized interventions and strategies to improve the overall well-being and health outcomes of cancer patients. The changes induced by PA may encourage an improved survival and lifespan, decreased mortality, and hopefully, reduce the strain induced by medical treatments.

## Figures and Tables

**Table 1:** Baseline characteristics of study participants: female breast cancer patients randomly divided into a Control Group (CG), where all subjects received the usual medical treatments, or an Exercise Group (EG), where subjects were additionally included in a 16-week online exercise training intervention. Values are presented as mean ± standard deviation (SD); BMI, body mass index; IPAQ, International Physical Activity Questionnaire; MET, metabolic equivalent; min, minutes; m, meter; Kg, kilogram; N.A., not applicable. *p*-value was determined with Student’s *t*-test by comparing each baseline characteristics between both groups (CG vs. EG).

	CG (*n* = 9)	EG (*n* = 14)	*p*-Value
Chronological age (years)	53.00 ± 6.65	50.21 ± 5.47	0.2852
Weight (kg)	61.13 ± 11.77	62.82 ± 6.39	0.6592
Height (cm)	163.2 ± 7.17	162.4 ± 7.45	0.8028
BMI (kg/m^2^)	22.76 ± 2.30	27.16 ± 6.11	0.0747
Type of surgery			
Quadrantectomy	5	8	N.A.
Mastectomy	4	5	N.A.
Medical treatments			
Chemo + hormonal + radio	2	3	N.A.
Hormonal + radio	4	7	N.A.
Hormonal	3	4	N.A.
Physical activity level			
IPAQ (MET-min/week)	834.6 ± 596.0	854.3 ± 587.5	0.9389

**Table 2:** Functional test analysis performed at the beginning (PRE) and at the end (POST) of the experimental protocol in female breast cancer patients, randomly assigned to the Control Group (CG, n = 9), where all subjects received the usual cancer treatments, or the Exercise Group (EG, n = 14), where volunteers were additionally included in a 16-week online training program. EG, Exercise Group; CG, Control Group; 6MWT, 6-min walking test; HGr, handgrip right; HGl, handgrip left; STS, sit-to-stand test; STR, sit and reach test; ScTr, scratch test right; ScTl, scratch test left. All data are presented as mean ± standard deviation (SD). *p*-value was determined with Student’s *t*-test of the comparison between PRE and POST within each group. Statistically significant *p*-values are in bold. Overall changes were calculated wither as the difference between POST and PRE (POST–PRE), indicated by ■, or by fold change, indicated by ▲.

		PRE	POST	*p*-Value	Overall Change
HGr	**CG:** **EG:**	29.30 ± 4.043 28.67 ± 3.584	28.91 ± 5.400 28.07 ± 3.984	0.1394 0.4983	0.8904 ± 0.2954 ▲
HGl	**CG:** **EG:**	24.12 ± 2.204 26.43 ± 3.995	20.80 ± 0.4967 25.43 ± 3.939	**0.0019** 0.3230	0.8563 ± 0.2939 ▲
STS	**CG:** **EG:**	17.44 ± 6.521 18.00 ± 3.843	18.43 ± 5.127 21.29 ± 4.906	0.3559 **0.0413**	1.032 ± 0.4021
STR	**CG:** **EG:**	2.278 ± 12.32 −0.5714 ± 9.277	2.571 ± 14.02 4.929 ± 8.265	0.3078 **0.0035**	3.952 ± 5.298 ■
ScTr	**CG:** **EG:**	25.22 ± 8.657 27.23 ± 9.418	22.00 ± 7.483 20.92 ± 7.609	0.33559 **0.0020**	−5 ± 5.944 ■
ScTl	**CG:** **EG:**	29.44 ± 11.95 27.46 ± 5.897	28.86 ± 12.85 22.77 ± 5.988	0.3559 **0.0002**	−2.947 ± 4.54 ■
6MWT	**CG:** **EG:**	533.2 ± 40.07 597.1 ± 67.05	486.4 ± 59.00 628.6 ± 63.20	0.1250 **0.0025**	1.017 ± 0.09696 ▲
Borg scale	**CG:** **EG:**	1.556 ± 0.7265 2.67 ± 0.4875	1.857 ± 0.8997 1.571 ± 1.072	0.5000 **0.0127**	−0.5 ± 1.36 ■

**Table 3:** Assessment of quality of life in female breast cancer patients randomly assigned to the Control Group (CG, n = 9), where all subjects received the usual cancer treatments; or Exercise Group (EG, n = 14), where volunteers were additionally included in a 16-week online training program. These measurements were made at the beginning (PRE) and at the end (POST) of the experimental protocol. *p*-value was determined with Student’s *t*-test of the comparison between PRE and POST within each group. Statistically significant *p*-values are in bold.

	PRE (Mean ± SD)	POST (Mean ± SD)	% Change	*p*-Value
EORTC QLQ C-30				
*Physical function* CG EG	90.01 ± 2.35 88.81 ± 7.04	91.21 ± 3.99 93.99 ± 4.01	+1.33 +5.83	0.323 **0.040**
*Emotional function* CG EG	88.23 ± 7.62 73.15 ± 11.52	87.61 ± 9.92 84.01 ± 21.11	−0.69 +14.84	0.498 0.342
*Cognitive function* CG EG	89.57 ± 12.01 85.08 ± 17.29	87.10 ± 10.30 87.41 ± 15.53	−2.76 +2.73	0.397 0.881
*Social function* CG EG	87.12 ± 13.95 82.78 ± 20.34	84.09 ± 13.99 92.09 ± 15.13	−2.55 +11.24	0.488 0.269
*Global health* CG EG	73.88 ± 10.90 64.66 ± 10.42	66.34 ± 21.92 71.44 ± 14.09	−10.21 +10.48	0.385 0.350

## Data Availability

The datasets used and/or analyzed during this study are available from the corresponding authors on reasonable request.

## References

[1] Kennedy B.J. (1988). Aging and Cancer. J. Clin. Oncol..

[2] López-Otín C., Blasco M.A., Partridge L., Serrano M., Kroemer G. (2013). The Hallmarks of Aging. Cell.

[3] Mandelblatt J.S., Ahles T.A., Lippman M.E., Isaacs C., Adams-Campbell L., Saykin A.J., Cohen H.J., Carroll J. (2021). Applying a Life Course Biological Age Framework to Improving the Care of Individuals With Adult Cancers: Review and Research Recommendations. JAMA Oncol..

[4] Rentscher K.E., Bethea T.N., Zhai W., Small B.J., Zhou X., Ahles T.A., Ahn J., Breen E.C., Cohen H.J., Extermann M. (2023). Epigenetic Aging in Older Breast Cancer Survivors and Noncancer Controls: Preliminary Findings from the Thinking and Living with Cancer Study. Cancer.

[5] Hurria A., Jones L., Muss H.B. (2016). Cancer Treatment as an Accelerated Aging Process: Assessment, Biomarkers, and Interventions. Am. Soc. Clin. Oncol. Educ. Book.

[6] Cupit-Link M.C., Kirkland J.L., Ness K.K., Armstrong G.T., Tchkonia T., LeBrasseur N.K., Armenian S.H., Ruddy K.J., Hashmi S.K. (2017). Biology of Premature Ageing in Survivors of Cancer. ESMO Open.

[7] Hodes R.J., Sierra F., Austad S.N., Epel E., Neigh G.N., Erlandson K.M., Schafer M.J., LeBrasseur N.K., Wiley C., Campisi J. (2016). Disease Drivers of Aging. Ann. N. Y. Acad. Sci..

[8] Ness K.K., Wogksch M.D. (2020). Frailty and Aging in Cancer Survivors. Transl. Res..

[9] Wang S., Prizment A., Thyagarajan B., Blaes A. (2021). Cancer Treatment-Induced Accelerated Aging in Cancer Survivors: Biology and Assessment. Cancers.

[10] Garagnani P., Bacalini M.G., Pirazzini C., Gori D., Giuliani C., Mari D., Di Blasio A.M., Gentilini D., Vitale G., Collino S. (2012). Methylation of *ELOVL*_2_ Gene as a New Epigenetic Marker of Age. Aging Cell.

[11] Li Y., Ma L. (2022). Relationship between Telomere Length and the Prognosis of Breast Cancer Based on Estrogen Receptor Status: A Mendelian Randomization Study. Front. Oncol..

[12] Paparazzo E., Lagani V., Geracitano S., Citrigno L., Aceto M.A., Malvaso A., Bruno F., Passarino G., Montesanto A. (2023). An ELOVL2-Based Epigenetic Clock for Forensic Age Prediction: A Systematic Review. Int. J. Mol. Sci..

[13] Kipling D. (1995). The Telomere.

[14] Nomikos N.N., Nikolaidis P.T., Sousa C.V., Papalois A.E., Rosemann T., Knechtle B. (2018). Exercise, Telomeres, and Cancer: “The Exercise-Telomere Hypothesis”. Front. Physiol..

[15] Okamoto K., Seimiya H. (2019). Revisiting Telomere Shortening in Cancer. Cells.

[16] Shammas M.A. (2011). Telomeres, Lifestyle, Cancer, and Aging. Curr. Opin. Clin. Nutr. Metab. Care.

[17] Cleal K., Norris K., Baird D. (2018). Telomere Length Dynamics and the Evolution of Cancer Genome Architecture. Int. J. Mol. Sci..

[18] Maciejowski J., De Lange T. (2017). Telomeres in Cancer: Tumour Suppression and Genome Instability. Nat. Rev. Mol. Cell Biol..

[19] Song S., Lee E., Kim H. (2022). Does Exercise Affect Telomere Length? A Systematic Review and Meta-Analysis of Randomized Controlled Trials. Medicina.

[20] Marques A., Gouveira É.R., Peralta M., Martins J., Venturini J., Henriques-Neto D., Sarmento H. (2020). Cardiorespiratory Fitness and Telomere Length: A Systematic Review. J. Sports Sci..

[21] Duan R., Fu Q., Sun Y., Li Q. (2022). Epigenetic Clock: A Promising Biomarker and Practical Tool in Aging. Ageing Res. Rev..

[22] Li Piani L., Vigano’ P., Somigliana E. (2023). Epigenetic Clocks and Female Fertility Timeline: A New Approach to an Old Issue?. Front. Cell Dev. Biol..

[23] Teschendorff A.E. (2020). A Comparison of Epigenetic Mitotic-like Clocks for Cancer Risk Prediction. Genome Med..

[24] Voisin S. (2021). Exercise and/or Stress Effects on the Epigenetic Clock. Stress: Genetics, Epigenetics and Genomics.

[25] Fransquet P.D., Wrigglesworth J., Woods R.L., Ernst M.E., Ryan J. (2019). The Epigenetic Clock as a Predictor of Disease and Mortality Risk: A Systematic Review and Meta-Analysis. Clin. Epigenet..

[26] Field A.E., Robertson N.A., Wang T., Havas A., Ideker T., Adams P.D. (2018). DNA Methylation Clocks in Aging: Categories, Causes, and Consequences. Mol. Cell.

[27] Horvath S. (2013). DNA Methylation Age of Human Tissues and Cell Types. Genome Biol..

[28] Horvath S., Raj K. (2018). DNA Methylation-Based Biomarkers and the Epigenetic Clock Theory of Ageing. Nat. Rev. Genet..

[29] Hannum G., Guinney J., Zhao L., Zhang L., Hughes G., Sadda S., Klotzle B., Bibikova M., Fan J.-B., Gao Y. (2013). Genome-Wide Methylation Profiles Reveal Quantitative Views of Human Aging Rates. Mol. Cell.

[30] Kabacik S., Lowe D., Fransen L., Leonard M., Ang S.-L., Whiteman C., Corsi S., Cohen H., Felton S., Bali R. (2022). The Relationship between Epigenetic Age and the Hallmarks of Aging in Human Cells. Nat. Aging.

[31] Levine M.E., Lu A.T., Quach A., Chen B.H., Assimes T.L., Bandinelli S., Hou L., Baccarelli A.A., Stewart J.D., Li Y. (2018). An Epigenetic Biomarker of Aging for Lifespan and Healthspan. Aging.

[32] Zbieć-Piekarska R., Spólnicka M., Kupiec T., Makowska Ż., Spas A., Parys-Proszek A., Kucharczyk K., Płoski R., Branicki W. (2015). Examination of DNA Methylation Status of the ELOVL2 Marker May Be Useful for Human Age Prediction in Forensic Science. Forensic Sci. Int. Genet..

[33] Zbieć-Piekarska R., Spólnicka M., Kupiec T., Parys-Proszek A., Makowska Ż., Pałeczka A., Kucharczyk K., Płoski R., Branicki W. (2015). Development of a Forensically Useful Age Prediction Method Based on DNA Methylation Analysis. Forensic Sci. Int. Genet..

[34] Li X., Wang J., Wang L., Gao Y., Feng G., Li G., Zou J., Yu M., Li Y.F., Liu C. (2022). Lipid Metabolism Dysfunction Induced by Age-Dependent DNA Methylation Accelerates Aging. Sig. Transduct. Target. Ther..

[35] Torregrosa C., Chorin F., Beltran E.E.M., Neuzillet C., Cardot-Ruffino V. (2022). Physical Activity as the Best Supportive Care in Cancer: The Clinician’s and the Researcher’s Perspectives. Cancers.

[36] Campbell K.L., Winters-Stone K.M., Wiskemann J., May A.M., Schwartz A.L., Courneya K.S., Zucker D.S., Matthews C.E., Ligibel J.A., Gerber L.H. (2019). Exercise Guidelines for Cancer Survivors: Consensus Statement from International Multidisciplinary Roundtable. Med. Sci. Sports Exerc..

[37] Rodríguez-Cañamero S., Cobo-Cuenca A.I., Carmona-Torres J.M., Pozuelo-Carrascosa D.P., Santacruz-Salas E., Rabanales-Sotos J.A., Cuesta-Mateos T., Laredo-Aguilera J.A. (2022). Impact of Physical Exercise in Advanced-stage Cancer Patients: Systematic Review and Meta-analysis. Cancer Med..

[38] Schmidt A., Cross G., Pitoia F. (2017). Metástasis a distancia en cáncer diferenciado de tiroides: Diagnóstico y tratamiento. Rev. Argent. Endocrinol. Metab..

[39] Ligibel J.A., Giobbie-Hurder A., Shockro L., Campbell N., Partridge A.H., Tolaney S.M., Lin N.U., Winer E.P. (2016). Randomized Trial of a Physical Activity Intervention in Women with Metastatic Breast Cancer. Cancer.

[40] Álvaro Sanz E., Abilés J., Garrido Siles M., Pérez Ruíz E., Alcaide García J., Rueda Domínguez A. (2021). Impact of Weight Loss on Cancer Patients’ Quality of Life at the Beginning of the Chemotherapy. Support. Care Cancer.

[41] Vangelov B., Venchiarutti R.L., Smee R.I. (2017). Critical Weight Loss in Patients With Oropharynx Cancer During Radiotherapy (± Chemotherapy). Nutr. Cancer.

[42] Albrecht T.A., Taylor A.G. (2012). Physical Activity in Patients with Advanced-Stage Cancer: A Systematic Review of the Literature. Clin. J. Oncol. Nurs..

[43] Sellami M., Bragazzi N., Prince M.S., Denham J., Elrayess M. (2021). Regular, Intense Exercise Training as a Healthy Aging Lifestyle Strategy: Preventing DNA Damage, Telomere Shortening and Adverse DNA Methylation Changes Over a Lifetime. Front. Genet..

[44] Światowy W.J., Drzewiecka H., Kliber M., Sąsiadek M., Karpiński P., Pławski A., Jagodziński P.P. (2021). Physical Activity and DNA Methylation in Humans. Int. J. Mol. Sci..

[45] García-Giménez J.L., Cánovas-Cervera I., Pallardó F.V. (2024). Oxidative Stress and Metabolism Meet Epigenetic Modulation in Physical Exercise. Free. Radic. Biol. Med..

[46] Voisin S., Eynon N., Yan X., Bishop D.J. (2015). Exercise Training and DNA Methylation in Humans. Acta Physiol..

[47] Moulton C., Murri A., Benotti G., Fantini C., Duranti G., Ceci R., Grazioli E., Cerulli C., Sgrò P., Rossi C. (2024). The Impact of Physical Activity on Promoter-Specific Methylation of Genes Involved in the Redox-Status and Disease Progression: A Longitudinal Study on Post-Surgery Female Breast Cancer Patients Undergoing Medical Treatment. Redox Biol..

[48] Fox F.A.U., Liu D., Breteler M.M.B., Aziz N.A. (2023). Physical Activity Is Associated with Slower Epigenetic Ageing—Findings from the Rhineland Study. Aging Cell.

[49] Jokai M., Torma F., McGreevy K.M., Koltai E., Bori Z., Babszki G., Bakonyi P., Gombos Z., Gyorgy B., Aczel D. (2023). DNA Methylation Clock DNAmFitAge Shows Regular Exercise Is Associated with Slower Aging and Systemic Adaptation. GeroScience.

[50] Kawamura T., Radak Z., Tabata H., Akiyama H., Nakamura N., Kawakami R., Ito T., Usui C., Jokai M., Torma F. (2024). Associations between Cardiorespiratory Fitness and Lifestyle-related Factors with DNA Methylation-based Ageing Clocks in Older Men: WASEDA’S Health Study. Aging Cell.

[51] Benitez-Buelga C., Sanchez-Barroso L., Gallardo M., Apellániz-Ruiz M., Inglada-Pérez L., Yanowski K., Carrillo J., Garcia-Estevez L., Calvo I., Perona R. (2015). Impact of Chemotherapy on Telomere Length in Sporadic and Familial Breast Cancer Patients. Breast Cancer Res. Treat..

[52] Pearce E.E., Alsaggaf R., Katta S., Dagnall C., Aubert G., Hicks B.D., Spellman S.R., Savage S.A., Horvath S., Gadalla S.M. (2022). Telomere Length and Epigenetic Clocks as Markers of Cellular Aging: A Comparative Study. GeroScience.

[53] Gallicchio L., Gadalla S.M., Murphy J.D., Simonds N.I. (2018). The Effect of Cancer Treatments on Telomere Length: A Systematic Review of the Literature. J. Natl. Cancer Inst..

[54] Wentzensen I.M., Mirabello L., Pfeiffer R.M., Savage S.A. (2011). The Association of Telomere Length and Cancer: A Meta-Analysis. Cancer Epidemiol. Biomark. Prev..

[55] Müezzinler A., Zaineddin A.K., Brenner H. (2013). A Systematic Review of Leukocyte Telomere Length and Age in Adults. Ageing Res. Rev..

[56] Levine M.E., Lu A.T., Chen B.H., Hernandez D.G., Singleton A.B., Ferrucci L., Bandinelli S., Salfati E., Manson J.E., Quach A. (2016). Menopause Accelerates Biological Aging. Proc. Natl. Acad. Sci. USA.

[57] Moulton C., Grazioli E., Antinozzi C., Fantini C., Cerulli C., Murri A., Duranti G., Ceci R., Vulpiani M.C., Pellegrini P. (2023). Online Home-Based Physical Activity Counteracts Changes of Redox-Status Biomarkers and Fitness Profiles during Treatment Programs in Postsurgery Female Breast Cancer Patients. Antioxidants.

[58] Joglekar M.V., Satoor S.N., Wong W.K.M., Cheng F., Ma R.C.W., Hardikar A.A. (2020). An Optimised Step-by-Step Protocol for Measuring Relative Telomere Length. Methods Protoc..

